# A Mixed Methods Process Evaluation of a Clustered-Randomized Controlled Trial to Determine the Effects of Community-Based Dietary Sodium Reduction in Rural China

**DOI:** 10.3389/fmed.2021.646576

**Published:** 2021-05-28

**Authors:** Hongling Chu, Jing Zhang, Michael D. Fetters, Wenyi Niu, Huijuan Li, Nicole Li, Lijing L. Yan, Yanfang Wang, Yangfeng Wu

**Affiliations:** ^1^Research Center of Clinical Epidemiology, Peking University Third Hospital, Beijing, China; ^2^The George Institute for Global Health at Peking University Health Science Center, Beijing, China; ^3^Department of Family Medicine and Mixed Methods Program, School of Medicine, University of Michigan, Ann Arbor, MI, United States; ^4^School of Public Health, Peking University Health Science Centre, Beijing, China; ^5^Peking University Clinical Research Institute, Beijing, China; ^6^George Institute for Global Health, University of New South Wales, Newtown, NSW, Australia; ^7^Global Health Research Center, Duke Kunshan University, Suzhou, China

**Keywords:** hypertension, clustered-randomized trial, salt reduction, complex intervention, process evaluation, mixed methods

## Abstract

**Purpose:** A clustered-randomized controlled trial was conducted to determine the effects of a sodium reduction program in 120 rural villages in Northern China. This mixed-methods process evaluation was used to investigate the implementation and to evaluate the feasibility of the complex intervention to translate the findings from clinical study to the real world.

**Methods:** A convergent mixed-methods process evaluation design was used in this study. Quantitative data were collected from activity logs and routine study records. Qualitative data were collected from 53 project stakeholders and 45 villagers from 10 intervention villages. Thematic analysis of qualitative interviews facilitated integration with the descriptive quantitative data analysis based on theory-informed domains of fidelity, delivery, reach, receipt, and contextual factors of intervention from a process evaluation framework.

**Results:** The intervention was implemented with high fidelity, delivery, reach, and receipt. A total of 5,450 sheets of posters, 31,400 calendars, and 78,000 sheets of stickers were delivered as planned, and 11 promotion activities were conducted in each village. Contextual factors hindering full uptake of the intervention included preference for salty taste, higher cost of low-sodium salt, and low education levels of villagers. Other contextual factors, positive policy support, administrative support, and staff enthusiasm were the facilitators for implementation.

**Conclusions:** This multifaceted intervention was implemented well and effectively in rural China. This process evaluation has indicated that conducting health education interventions in rural areas requires policy and administrative support, enthusiastic staff, easy-to-understand health education materials and activities, and key persons, but tempered expectations as behavior change requires time. This project demonstrates the feasibility and benefits of using mixed-methods process evaluation in large-scale studies.

## Introduction

Stroke is the leading cause of death in China, responsible for about 1.7 million deaths each year (1, 2). Excess sodium intake is a key determinant of high blood pressure (3), the leading cause of stroke (4). The magnitude of the effect of sodium on blood pressure is such that each 75-mmol difference in daily salt intake translates into an ~5.4 mm Hg difference in systolic blood pressure among individuals with hypertension, and 2.4 mm Hg among individuals without hypertension (3). Chinese people, especially those living in northern rural areas, have the highest sodium intake levels in the world, where hypertension and the incidence of stroke are also all very high (5). In Western populations, most dietary sodium derives from processed and restaurant foods, but in rural China the major source comes from salt and condiments added in home cooking (6).

Given this hypertensive crisis in rural China, a clinical trial was conducted to identify a novel, low-cost, scalable, and sustainable, community-based strategy for the prevention of blood pressure-related diseases in rural China (7). The trial was registered with clinicaltrial.gov in December 2010, registration number NCT01259700.

The main study was published (8). The overall intervention was designed on the basis of the health belief model (HBM). The HBM is a theoretical model that has been constructed from six domains, which are perceived susceptibility, severity, barriers, benefit, cues to action, and self-efficacy. According to the framework of the HBM and proved cost-effective strategy for sodium reduction (9), the complex intervention contained three parts, health education materials with key message about salt reduction (posters, calendars, and stickers were pasted, respectively, on the wall outside, indoors, and in salt containers), health education activities (launch events, activities organized in consideration of local context, activities for individuals at elevated risk of cardiovascular disease, and student-to-parent education activities), and low-sodium salt substitute supply (Table 1). The intervention is further depicted in the tables in the appendix (Supplementary Material in Appendix 1).

**Table 1:** The health belief model and derived intervention used in this project.

**Domains of HBM**	**Features of the intervention in this project according to the HBM**	**Intervention**
**1. Perceived susceptibility, severity**	**Improving the awareness of:** a. How much salt is consumed b. High salt intake worsens health and diseases caused by high salt intake	**a. Health education materials** - Posters- Calendars - Stickers for placing on salt containers
**2. Perceived benefit, barriers, cues to action**	**Providing the information and cues about:** a. The benefits of consuming less salt b. The effects of low-sodium salt c. Removing concerns that low salt intake would result in no energy to work. d. To create a salt-reduction atmosphere and tell them how to reduce salt intake (knowledge and behaviors)	**b. Health education activities** - Program launch events - Activities organized in consideration of local context - Activities for individuals at elevated risk of cardiovascular disease - Student-to-parent education activities
**3. Perceived self-efficacy**	**Organizing activities to help them improve their self efficacy:** a. To carry out activities to encourage their beliefs and behaviors for salt reduction	**c. Low-sodium salt substitute supply**

After complex intervention being conducted for 18 months, 1,903 people had valid 24-h urine collections. The mean urinary sodium excretion in intervention compared with control villages was reduced by 5.5% (−14 mmol/day, 95% confidence interval −26 to −1; *p* = 0.03). In the intervention group, potassium excretion was increased by 16% (+7 mmol/ day, +4 to +10; *p* < 0.001), and the sodium to potassium ratio declined by 15% (−0.9, −1.2 to −0.5; *p* < 0.001). Between the intervention and control groups, the mean blood pressure differences were −1.1 mm Hg systolic (−3.3 to +1.1; *p* = 0.33) and −0.7 mm Hg diastolic (−2.2 to +0.8, *p* = 0.35). The difference in the proportion with hypertension was −1.3% (−5.1 to 2.5, *p* = 0.56) (8). The absence of effects on blood pressure reflects the moderate changes in sodium and potassium intake achieved (Supplementary Material in Appendix 1).

However, alongside intervention efficacy, some questions are still unclear, these are as follows: how are the components of the complex intervention implemented; what are the barriers or facilitators within its context; and how can implementation be optimized in future practice. Therefore, the mixed-methods approach was used to integrate qualitative and quantitative aspects of this study, to investigate the fidelity, delivery, reach, receipt, and context of intervention. The current study could provide us a comprehensive view of implementation and effectiveness and promoted evidence-based practice for village doctors who provide the basic medical service as a family doctor to prevent and control chronic disease in local village in rural China.

## Methods

### Theoretical Framework of Process Evaluation

“How-to Guide” (10) and REAIM frameworks (11) were used for the process evaluation to examine the level of implementation comprehensively. This involves understanding the implementation of intervention in terms of (1) fidelity, defined as to what extent the intervention was implemented consistently with the underlying theory as planned; (2) delivery, defined as to what extent all of the intended activities, training, and materials were provided to program participants; (3) reach, defined as the absolute number, proportion, and representativeness of individuals who are willing to participate in a given initiative, intervention, or program; (4) receipt, defined as to how participants reacted to specific aspects of the intervention; and (5) context, defined as what contextual factors influence implementation or the intervention outcome.

### Design

**Figure 1:**
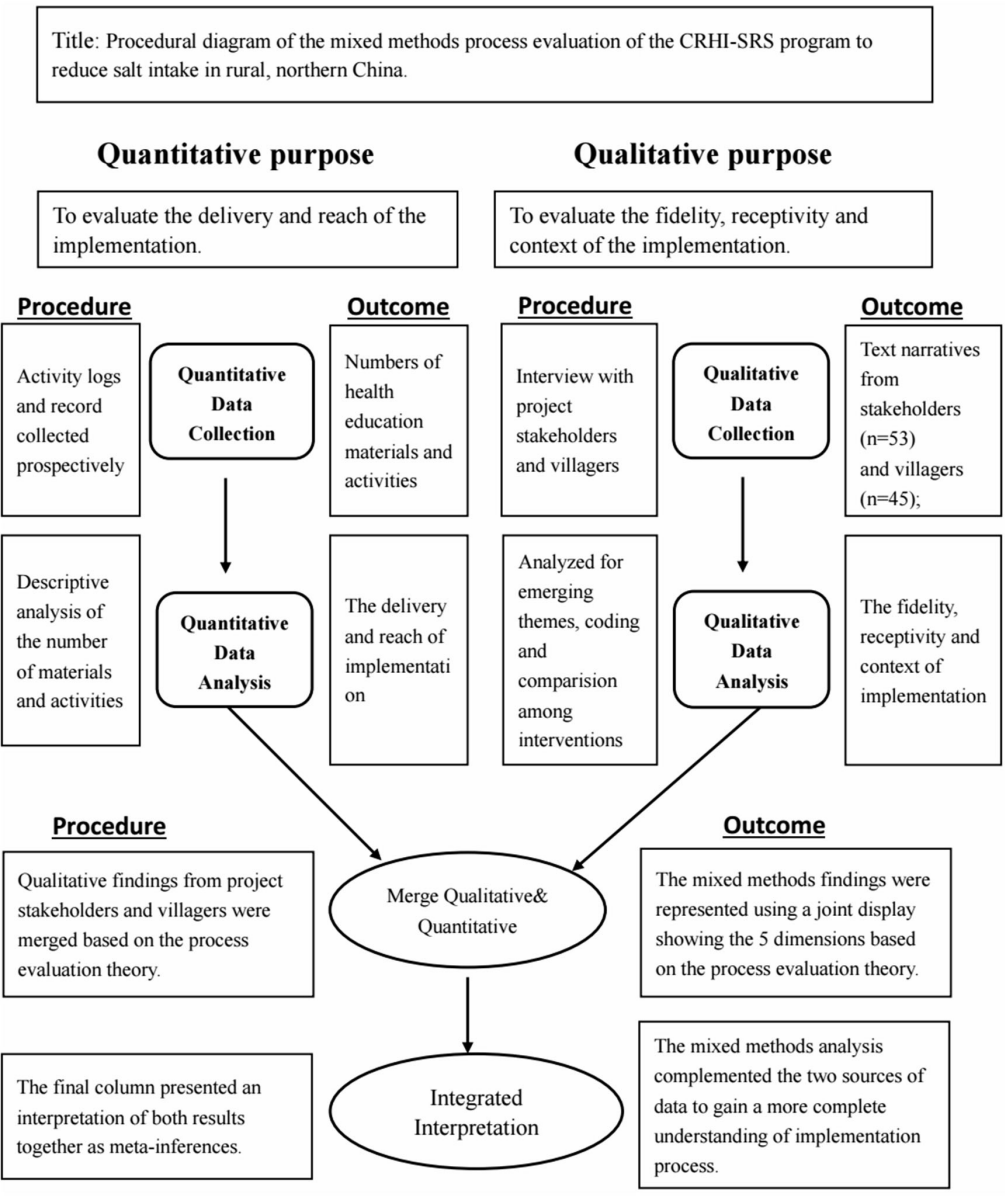
Diagram of convergent mixed-methods process evaluation.

A convergent mixed-methods design (12) was used in the current study that was guided by the process evaluation theoretical framework. While data were collected prospectively, the analysis of the data occurred at the same time after the intervention. The impetus for the mixed-data evaluation was to provide a comprehensive view of the extent the intervention worked, and to understand why the interventions conducted in this study were effective (Figure 1).

### Study Settings and Participants

The main trial was conducted in five Northern Provinces of China, including Hebei, Liaoning, Ningxia, Shanxi, and Shaanxi (7). Two counties from each province were selected for participation; twelve townships of each county were engaged with total 120 to wnships. A typical township in the study comprised ~17 villages with a total population of 25,000. There was one village doctor in each village who provides basic health services there. The average population of included villages was 1,867 with 512 households. The annual per capita income was $884, and 91.5% participants have <9 years of education. The village doctors, who conducted intervention in this study, have played a significant role in providing basic health services to villagers when the area had great shortage of health resources.

### Data Collection

The health education activities and process evaluation were launched in May 2011, and health education activities ended on September 2012, while the process evaluation continued throughout the project and afterward until March 2013. The study data sources are represented in Supplementary Material in Appendix 1.

### Quantitative Data

Data about delivery of the materials were collected from 150 activity logs and 91 routine records recorded prospectively by the county project officers and project assistants.

### Qualitative Data

The strategy of purposeful sampling was used to select participants. Using a semi-structured, face-to-face depth interview (interview guides are shown in Appendix 2 in Supplementary Material), the qualitative evaluation was conducted in 10 intervention villages from five provinces (two villages intentionally sampled in each county, one county from each province). Interviews were conducted among 48 key stakeholders including five provincial project investigators, five province project coordinators, five county project officers, five county project governors, five county health educators, eight town health educators, 15 village doctors; and 45 villagers from intervention villages including 19 housewives, 13 general villagers, and 13 villagers with a high risk of cardiovascular disease (Supplementary Material in Appendix 1).

### Data Analysis

#### Intramethod Data Analysis

The number and percentage of the education materials distributed and health education events held were calculated for each component on three dimensions including fidelity, delivery, and dose reached to villagers. SPSS 24.0 (IBM SPSS Statistics for Windows, Version 24.0. Armonk, NY: IBM Corp)software was used to analyze the quantitative data. An immersion/crystallization approach was used to analyze the qualitative data based on the dimension of receipt and contextual factors (13). The transcribed data were analyzed using Nvivo 11 (QRS, Australia) software of Chinese version. Three researchers (CH, LH, and GW) coded and analyzed the transcripts by making sense of the transcribed data, developing codes, categorizing the data, and abstracting. We discussed every discrepancy regarding the finding interpretation until reaching consensus. The analysts calibrated the coding process after reviewing one independently coded interview. Models were constructed through the analysis and then confirmed by reviewing the interview transcripts. The final agreement was achieved through the review of entire research team.

#### Mixed Data Analysis

The mixed data were analyzed based on the study process evaluation framework using the process of joint display analysis (14). This iterative process of juxtaposing links quantitative and qualitative data together, examining the implications of each other and looking for new facilitates drawing conclusions in light of both types of findings.

## Results

Interviews were completed among 93 stakeholders, and 150 records were collected prospectively. We present the quantitative findings for the three dimensions of process evaluation of fidelity to protocol, delivery and reach, and qualitative findings for two dimensions of receipt and contextual factors. The interview responses have been summarized in the results and specific quotations can be found in the related sections.

### Fidelity/Delivery

Fidelity was determined by comparing the activities outlined in the strategic action plan with delivered activities. This comparison showed that all health education materials and activities were implemented as planned, with high fidelity (Supplementary Material in Appendix 1). A total of 5,450 sheets with five different types of posters, 62,800 sheets of yearly calendars, and 78,000 pieces of special designed stickers were delivered to intervention villages. Seven hundred and twenty health education events were held in total, and 115, 228 packs of salt substitute were delivered to the village stores.

In terms of student-to-parent education activities, the numbers of planned and delivered were 60 and 120, respectively (Table 2). The reason for this was when we completed the first round of student education activities (60 events), we found that the students were willing to participate in activities and the effect of health education was relatively good. Therefore, the second round of activities (60 events) was conducted. From qualitative interviews, some interviewees mentioned that children and primary school kids paid more attention to salt reduction, “I found that children were interested in those pictures, stories on the calendar, as well as the activities.” (Villager).

**Table 2:** Joint display of fidelity, delivered, reach, receipt, and meta inferences by each component of intervention.

**Dimension**	**Posters**	**Calendars**	**Stickers for placing on salt containers**	**Program launch events**	**Activities organized in consideration of local context**	**Activities for individuals at elevated risk of cardiovascular disease**	**Student-to-parent education activities**	**Low-sodium salt substitute supply**
**Fidelity** (to protocol)	High	High	High	High	High	High	Moderate	High
**Delivery** (planned/delivered)	5,450/5,450 units	31,400/31,400 units	78,000/78,000 units	60/60 events	300/300 events	240/228 events	60/120 events	NA/115, 228 bags
**Reach**	40/45 interviewees	40/45 interviewees	39/45 interviewees	43/45 interviewees	22/32 interviewees	12/13 interviewees	1,595 children's worksheets	37/45 interviewees
**Receipt**	Being popular, easy-to-understand, and simple	Deemed practical	Served to remind when using salt	Created a specific and suitable atmosphere for salt reduction	Some villagers felt the content became repetitive and wanted more variety of information	Some elders or people with CVD could not participate	Primary school students were interested in these activities	Some felt the taste was less salty than regular salt, and it was more expensive
**Meta inferences**	Recommended and accepted by villagers.	Recommended	Recommended	Strongly recommended	Strongly recommended	Moderately recommended	Strongly recommended	Recommended

### Reach

The data was transformed from the interviews. It showed that the proportion of attended health education activities was low (61.5%) in general villagers, while the number was relatively high (92.3%) for people with high risk of cardiovascular diseases (Table 3). From the qualitative interviews, we found that a dearth of young adults participated in activities due to out-migration to cities. “*There are many middle-aged and young people moved into the urban cities or temporarily work there, who were unable to participate.” (VHE)*. In addition, some villagers felt that the content later during implementation became repetitive. “*There was no new ideas added with passing of time.” (CHE)*. These two reasons might explain a low reach of the health education activities in the general villagers.

**Table 3:** Reach of each component that interviewees exposed to the intervention.

**Interview groups and number interviewed**	**Generalvillagers** ***N =* 13**	**Housewife** ***N =* 19**	**Villagers at elevated risk of CVD *N =* 13**	**Total** ***N =* 45**
	**n (%)**	**n (%)**	**n (%)**	**n (%)**
Heard of this project	12 (92.3)	19 (100)	12 (92.3)	43 (95.6)
Saw posters	11 (84.6)	18 (94.7)	11 (84.6)	40 (88.9)
Received calendars	10 (76.9)	17 (89.4)	13 (100)	40 (88.9)
Received stickers	11 (84.6)	17 (89.4)	11 (84.6)	39 (86.7)
Attended health education activities	8 (61.5)	14 (73.7)	12 (92.3)	43 (95.6)
Used a low-sodium salt substitute	10 (76.9)	15 (68.4)	12 (92.3)	37 (82.2)

### Receptivity of Intervention

Generally, the salt reduction project was accepted and the local people were satisfied. The health education materials, including posters, calendars, and stickers, were accepted by the local people because they were popular, simple, easy-to-understand, and practical. However, posters placed outdoors were easily lost and less effective than posters placed indoors. Some interviewees suggested writing slogans on walls.

The health education activities created a specific and suitable atmosphere important during program launch and provided the detailed information and cues necessary to reduce salt intake. Some interviewees mentioned that children and primary students paid more attention to salt reduction. However, some villagers felt that the content later during implementation became repetitive and wanted a greater variety of information. Interviewees had many valuable suggestions, such as leading the salt reduction activities by enthusiastic volunteers who are the elders or children, and that activities should avoid busy farming times.

In terms of low-sodium salt substitute supply, most interviewees deemed that low-sodium salt substitute is acceptable because the village doctor told them that it is healthier than regular salt, even though some felt that the taste was less salty than regular salt and that it was more expensive. Supplementary Material in Appendix 3 provides typical interview quotes regarding receipt of the intervention.

### Contextual Factors

Contextual factors influencing implementation included policy support, administrative support, staff enthusiasm, and out-migration of young adults to cities. At present, some national chronic disease policies have been implemented in certain areas and incorporated into public health service projects. From the interviews, we serendipitously found that some areas with policy support for a chronic disease project improved the conduct of this project due to interactive and mutual support of both projects. Administrative support, in general, can improve the quality of project implementation. However, in some cases, there are some staff who conducted project work only because of administrative pressures—this has potentially negative implications for the quality of the project. Health educators paid time and effort on the project varying from 10 to 70%. In most counties, the health educators had more than one job. Thus, in addition to the program work, they already had a very heavy workload. This led to time conflicts and hampered the quality of the program's implementation. Still, some health educators prepared for this project in the evening or on weekends. This included drafting reports and other administrative activities. Some experienced health educators conducted a variety of health education activities. There was a dearth of young adults participating in activities due to out-migration to cities.

Contextual factors influencing the intervention's effect included the following: adding too much salt when cooking and eating pickled foods as long-standing behaviors, change is a long-term process that requires gradual adaptation; and as the education level of villagers is generally low, it takes a long time to change and maintain healthy behaviors. The Supplementary Material in Appendix 4 provides quotes of contextual factors from the study interviews.

To integrate the quantitative and qualitative results, we created Table 2 based on the process evaluation framework and the structure of a joint display.

## Discussion

The clustered-randomized controlled trial was a rigorously conducted trial. The process evaluation found that the fidelity, delivery, reach, and receipt of the intervention were high. Posters were recommended for the value of creating a positive atmosphere; calendars were practical as they could be posted on the wall of the house for a long time and provide reminder information. Stickers for placing on salt containers were recommended as they prompted caution when using salt during cooking. This is especially important, since sodium intake primarily comes from added salt during cooking in the study population (6).

A program launch event is strongly recommended as it captured the attention of most villagers and implementers and created an atmosphere conducive to salt reduction among the whole village. Activities organized in consideration of the local context are also strongly recommended as they maximized the involvement of villagers in the sodium reduction intervention by holding a variety of activities based on local culture. Activities for high-risk individuals were only moderately recommended as they are very time consuming and covered only fewer topics about health, while the targeted people felt less benefit. The principle of “simple and repetitive” in health education in this case was not found suitable for activities (15). Villagers need more health-related knowledge, not simply a repetition of the same information. Interestingly, the student-to-parent education activities are strongly recommended because students played an influential role in families and they readily adapted the healthy behavior. From the interview, we also find that schoolchildren could take a leadership role in the sodium reduction intervention because of their key role in the family. This finding is seen also in a sequential school-based education program to reduce salt intake in children and their families (16, 17).

Notably, there were contextual factors influencing implementation and/or intervention effectiveness. Policy support, administrative support, and staff enthusiasm were all found to be facilitators, as they enhanced implementing this complex intervention in rural China. In contrast, lack of an effective governance mechanism to implement in low- and middle-income countries (LMICs) has been identified as an ongoing challenge in previous research (18, 19). The current project involves village doctors who conduct implement intervention as they easily gain trust from local villagers.

Moreover, there were three main barriers found from process evaluation. The first one is that general villagers were involved in the survey at the end of the intervention, although general villagers had a low reach in health education activities during intervention. This might make the evaluated effect lower than the actual effect. Secondly are adding too much salt when cooking, and eating pickled foods, which were long-standing dietary habits. It is well-known that behavior change is a long-term process requiring gradual adaptation. Many studies have identified that the strong cultural importance of feasting and people's perception of salty food taste were barriers of sodium intake reduction (20). Third, because of the low educational level of villagers, it might take a longer time to change and maintain healthy behavior (21). This also explains the secondary outcome that over 18 months' intervention, blood pressure did not differ significantly between the intervention and control groups (8).

### Limitations and Strengths

As for limitations, a. the evaluation would have been further strengthened if some process measurements could have been collected after completion of investigation, e.g., reach for each component of the intervention; b. data were not intentionally collected to show why differences in the extent of implementation had varied outcomes among different villages, because the process evaluation was conducted before the study outcomes were analyzed; and c. the insights about receptivity of intervention and context of this study from different populations including health educators, villagers, and implementers will be further analyzed for future research. As for strengths, the mixed-data evaluation provided a comprehensive understanding of the extent of the implementation and mechanism of effectiveness. It also promoted evidence-based practices in primary healthcare systems in rural China.

## Conclusion

When conducting health education interventions for salt reduction in rural China, the design of easy-to-understand educational materials including posters, calendars, and stickers proved to be effectively implemented ways to attract the attention of villagers. The findings suggest that interventions deeply involving primary school students or enthusiastic volunteers can promote the implementation of health education activities. Stocking salt substitutes in stores appears to provide convenient access for villagers. Meanwhile, policy and administrative support and staff with enthusiasm were important for the success of the intervention. These recommendations may be relevant in other low-middle-income countries. This project also demonstrates the feasibility and benefits of using a mixed-methods process evaluation in large-scale clinical trials and promotes evidence-based practices for village doctors who provide the basic medicine care and promote behavior change as a family doctor to prevent and manage chronic disease in rural China. This successful example may also help in the adoption of mixed-methods health service research in China.

## Prior Presentation

1. The 18th National Annual Conference of Clinical Epidemiology and Evidence-based Medicine, Aug. 24, 2019, Dalian, China.
2. MMIRA Asia Regional Conference, Sept. 15, 2019, Hamamatsu, Japan.

## Data Availability Statement

The raw data supporting the conclusions of this article will be made available by the authors, without undue reservation.

## Ethics Statement

The studies involving human participants were reviewed and approved by Ethics Committee of the Peking University Health Science Center. The patients/participants provided their written informed consent to participate in this study.

## Author Contributions

YWu, MF, and YWa: conceptualization. NL, LY, YWu, HL, WN, MF, and JZ: writing—review and editing. HC, JZ, MF, and YWa: writing—original draft. HC: software. HC, JZ, and HL: project administration and formal analysis. YWa, WN, YWu, LY, NL, HC, and HL: methodology. YWu, LY, NL, WN, and JZ: investigation. YWu: funding acquisition. All authors contributed to the article and approved the submitted version.

## Conflict of Interest

The authors declare that the research was conducted in the absence of any commercial or financial relationships that could be construed as a potential conflict of interest.
