# Supplementary material for: A Mixed Methods Process Evaluation of a Clustered-Randomized Controlled Trial to Determine the Effects of Community-Based Dietary Sodium Reduction in Rural China
Source: Front Med (Lausanne). 2021 May 28;8:646576. doi: 10.3389/fmed.2021.646576 (PMC8192799; doi:10.3389/fmed.2021.646576)
Supplement: Supplementary file 1 [file Data_Sheet_1.docx]

**Appendix 1**

**Table 1. Health education materials: posters**

| **Type of materials** | **Poster 1** | **Poster 2** | **Poster 3** | **Poster 4** | **Poster 5** |
| --- | --- | --- | --- | --- | --- |
| Pictures | 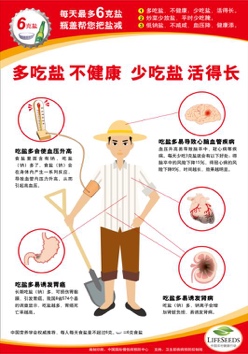 | 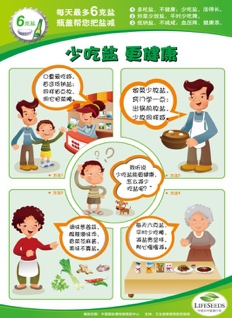 | 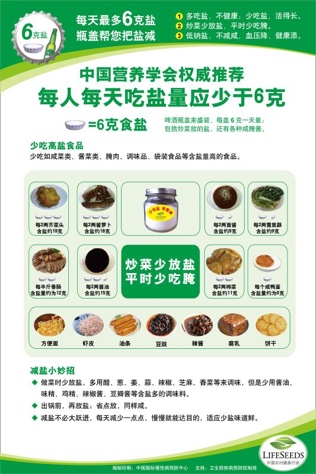 | 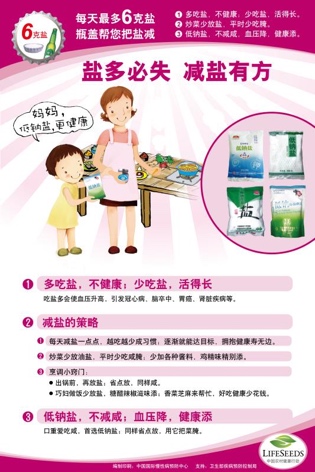 | 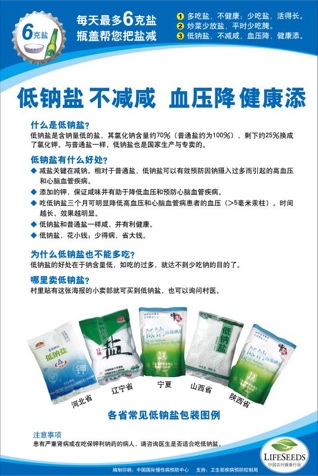 |
| Key point | Risks of excess sodium intake | Benefits of low sodium intake | Recommended daily salt intake level, and recommending less processed food, less cured meat/pickles, etc.) | Tips for eating less salt and how to reduce sodium intake | Benefits of using low sodium salt |

**Table 2. Health education materials: calendars and stickers**

| Type of materials | Calendar 1 | Calendar 1 | Sticker for placing on salt containers 1 | Sticker 2 for placing on salt containers |
| --- | --- | --- | --- | --- |
| Pictures | 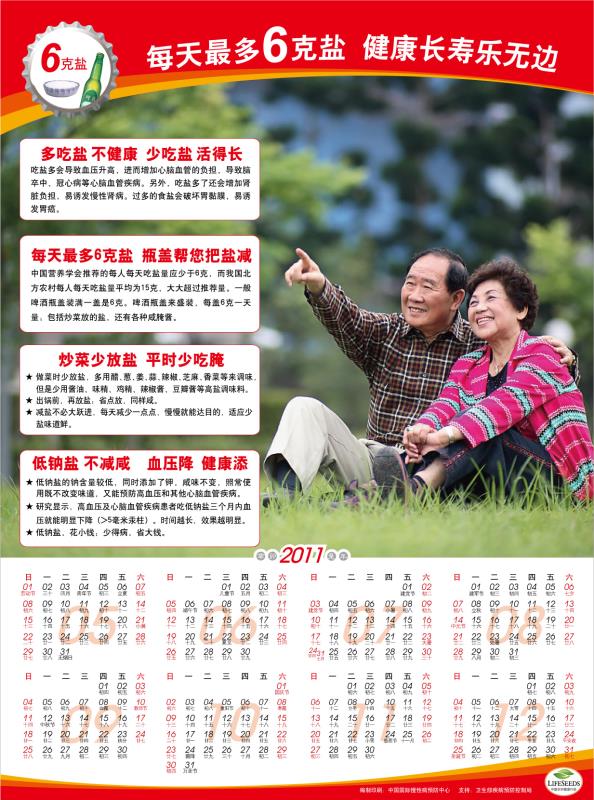 | 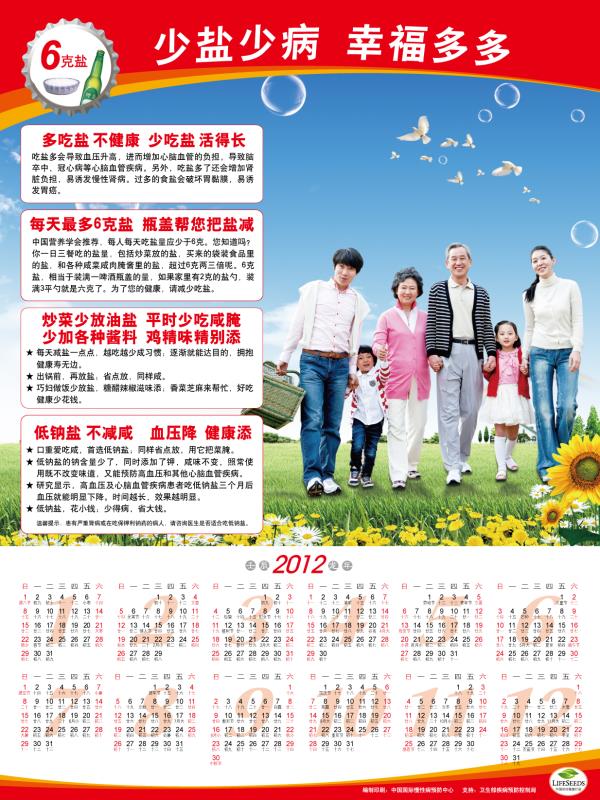 | 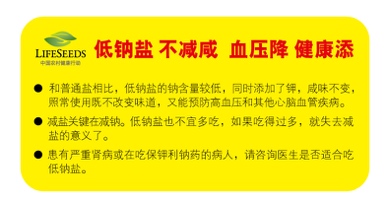 | 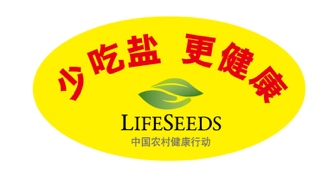 |
| Key point | All key messages about sodium reduction | All key messages about sodium reduction | Benefits of low sodium salt | “Less salt, better health” |

**Table 3. Introduction of activities**

| **Activities** | **Aim** | **Overview** | **Image** |
| --- | --- | --- | --- |
| Program launch events | To create an atmosphere that appeals to the whole village and have a discussion about salt reduction | At the beginning of the salt reduction program, a program launch event was organized to introduce the program and basic knowledge about salt intake to villagers. In addition to the villagers, the CHE, THE and VD were required to attend. | 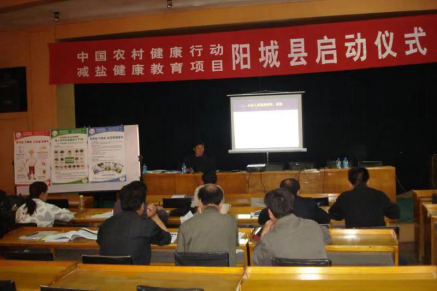 |
| **Activities organized in consideration of local context** | To maximize the involvement of villagers in the sodium reduction intervention by holding a variety of activities based on local culture. | Recognizing that culture varies among different villages, village health educators organized activities about sodium reduction that were combined with local activities or cultural events such as the Shanxi Opera or other local health education activities. | 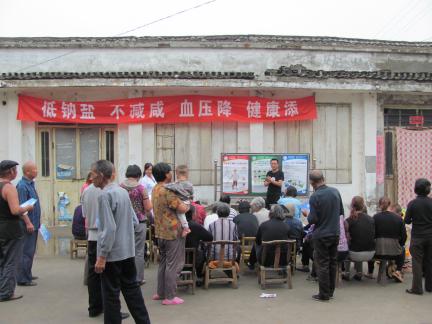 |
| **Activities for individuals at elevated risk of cardiovascular disease** | To focus on individuals at elevated risk of cardiovascular disease as they are in most need of salt reduction, and can gain the most benefit from sodium intake reduction. | To reach individuals at high risk of cardiovascular disease, the intervention used a lecture format to emphasize the importance of salt reduction and how to control salt intake. | 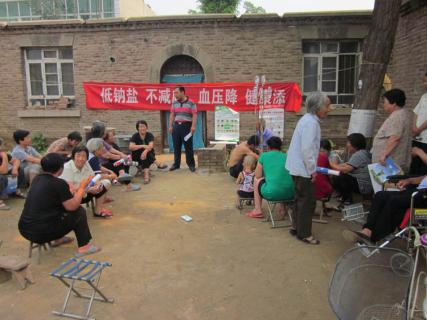 |
| **Student-to-**  **parent education activities** | To have students lead sodium reduction activities in their families, because students play an influential role in their families. | Health educators encouraged students to participate in salt reduction activities with their families, and to complete an educational worksheet on reducing dietary salt intake. | 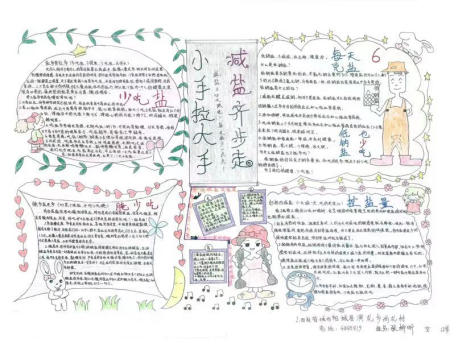 |
| **Low sodium salt substitute supply** | To provide convenient access to low sodium salt substitute for villagers | Coordinating the local product enterprise to assist the project implementer to deliver low sodium salt substitute to every store in each intervention village. | 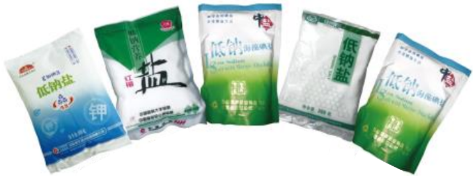 |

**Table 4. The main result of trial from the article published before**

Estimated effects of sodium reduction strategy for 60 intervention compared to 59 control villages.


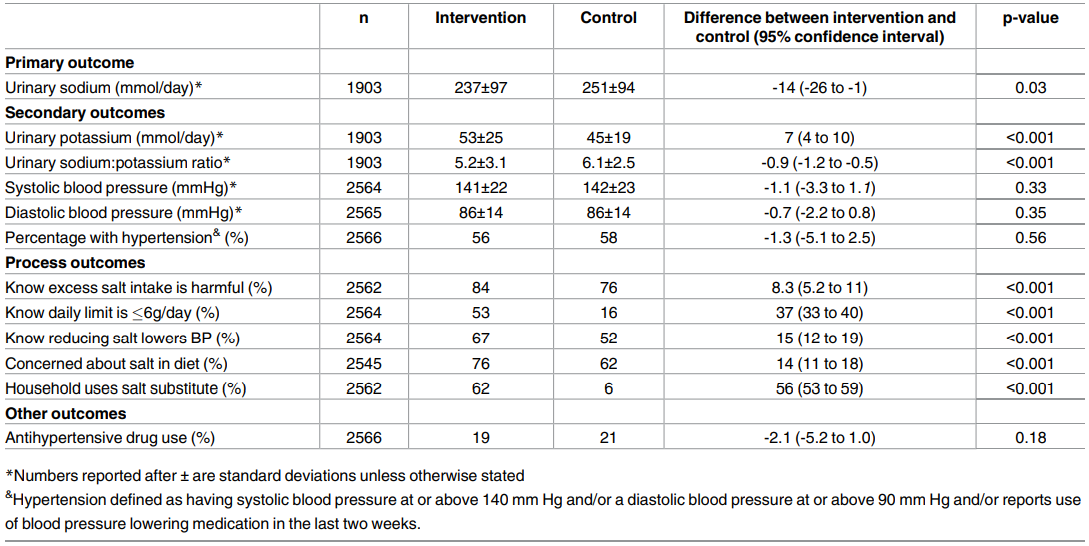


**Table 5. Data sources**

| **General Type?** | **Who/What?**  **(Sampling Strategy)** | **How Many?** | **Comments** |
| --- | --- | --- | --- |
| **Quantitative Data Sources** | | | |
| **Activity logs-** collected prospectively by county project officer on: 1) delivery of training of village health educators and 2) delivery of health education activities | -- CPO assessments (n=10) of the fidelity of the training of the village health educator according to the study protocol  -- Dates the health education activities were conducted (n=110 events)  -- CPO assessments of the fidelity of the health education activities by the local health educators according to the study protocol (n=648 health education activities) | --County Project Officer (CPOs)  (n=10)  --Health Educators  (n=60)  --Intervention villages  (n=60) | 4 types of health education activities for each intervention village:  1) Initiative activities (n=60)  2) Activities organized according to local context (n=300)  3) Activities for individuals at elevated risk of cardiovascular disease (n=228)  4) Student-to-parent education activities (n=120) |
| **Routine records-** collected prospectively by project assistants to document the delivery of health education materials | Type and number of health education materials distributed to 60 villages  -- posters (n=5450)  -- calendars (n=31,400)  -- stickers (n=78,000) | -- Project assistants  (n=2)  -- Intervention villages (n=60) | Village health educators distributed  -- 5 types of posters in the villages  -- Calendars and stickers house to house  -- Number actually delivered depended on the size of village |
| **Qualitative Data Sources** | | | |
| **Province-level semi-structured, face-to-face depth interviews** | All province level members of the project team from 5 provinces    Sampling strategy: purposeful sampling to represent all key stakeholders | PPI n=5  PPC n=5  CPO n=5  CPG n=5  CHE n=5  THE n=8  VD n=15  Total N=48 | The primary goal was to assess the fidelity and context of the intervention.    Project team members included all of the main grant participants. |
| **Village level semi-structured, face-to-face depth interviews** | Villagers in the intervention  2-5 per province  Criterion sampling:  -Women who were the primary cook of the family in villages;  -General villagers;  -Villagers with a high risk of cardiovascular disease | HW n=19  GV n=13  VHRC n=13  Total N=45 | The primary goal was to assess the experiences of villagers during the intervention.  The qualitative findings were transformed into quantitative data. |

**Table 6. Participants in qualitative interviews**

| **Participant Title** | **Abbreviation** | **Role/responsibilities** | **Number**  **N=93** | **Interview guide** |
| --- | --- | --- | --- | --- |
| Provincial Project Investigator | PPI | Responsible for overseeing the project and implementation in each province | n=5 | Interview guide 1 for project managers at province and county level.  (Appendix 2) |
| Provincial Project coordinator | PPC | Assists the PI: instructs the PO to implement the intervention according to the study protocol; and acts as a communicator with the Beijing project center | n=5 |  |
| County Project Officer | CPO | Responsible for communicating with others involved in project | n=5 |  |
| County Project Governor | CPG | Provides policy support for project implementation | n=5 |  |
| County Health Educator | CHE | Responsible for communicating with village health educators, and organizing training for health educators | n=5 | Interview guide 2 for health educators at different level.  (Appendix 2) |
| Town Health Educator | THE | Conducting health education activities with village doctors | n=8 |  |
| Village Doctor | VD | Conducting health education activities | n=15 |  |
| Housewife | HW | Responsible for cooking in a family | n=19 | Interview guide 3 for villagers.  (Appendix 2) |
| General Villagers | VG | General villagers without a high risk of CVD and who also do not cook for others | n=13 |  |
| Villagers with High Risk of CVD | VHRC | Villagers with a high risk of cardiovascular disease | n=13 |  |

**Table 7. Fidelity/delivery by intervention component**

| items | Planned time | Implemented  time | Numbers of health materials distributed or events implemented |
| --- | --- | --- | --- |
| Health materials |  |  |  |
| Poster 1 | 2011.05 | 2011.07 | 970 |
| Poster 2 | 2011.05 | 2011.07 | 970 |
| Poster 3 | 2011.05 | 2011.07 | 1170 |
| Poster 4 | 2011.07 | 2011.11 | 1170 |
| Poster 5 | 2011.08 | 2011.11 | 1170 |
| Calendar 1 | 2011.05 | 2011.05 | 31400 |
| Calendar 2 | 2012.01 | 2011.12 | 31400 |
| Sticker 1 | 2011.05 | 2011.05 | 31400 |
| Sticker 2 | 2012.01 | 2011.12 | 46600 |
| Health education activities |  |  |  |
| Program launch event | 2011.05 | 2011.05.18-2011.06.04 | 1 time per village，60 events in total |
| Activities organized in consideration of local context | 2011.06-  2012.09 | 2011.06.22-2012.09.24 | 5 times per village，300 events in total |
| Activities for individuals at elevated risk of cardiovascular disease | 2011.06-  2012.08 | 2011.06.18-2012.08.30 | 4 times per village，240 events in total |
| Student-to-parent education activities | 2011.07 | 2011.07-2012.07 | 2 times per village，120 events in total |
| Low sodium salt substitute supply | 2011.05-  2012.09 | 2011.05-2012.09 | 115, 228 bags |
