# Supplementary material for: A Mixed Methods Process Evaluation of a Clustered-Randomized Controlled Trial to Determine the Effects of Community-Based Dietary Sodium Reduction in Rural China
Source: Front Med (Lausanne). 2021 May 28;8:646576. doi: 10.3389/fmed.2021.646576 (PMC8192799; doi:10.3389/fmed.2021.646576)
Supplement: Supplementary file 2 [file Data_Sheet_2.doc]

**Appendix 2. Interview guides**

**Interview guide 1. Project stakeholders at the province and county level.**

1. Is there any policy or projects about cardio-cerebral vascular disease prevention and control implementation in this province? Specifically, how are these projects implemented? (frequency, scope, content, etc.) In your opinion, what are the barriers or benefits of this project? Why？
2. What is your overall impression of this project? What are the factors that impact the implementation or results? What advice do you have for improving these interventions?
3. What problems have been discovered during the implementation of the project? How to solve it?
4. What has been the impact of local customs and geography (such as geographical location, economic conditions, village dietary, culture, education levels, etc.) on project interventions.

**Interview guide 2. Health Educators at county, township and village levels.**

1. Are you responsible for work besides providing health education? How much time (percentage) did this project take up of your work? Were there any conflicts between your other work and this project? If there was a conflict, how did you usually solve it?
2. What is your overall impression of this project? What are the factors that impact the implementation or results? What advice do you have for improving this intervention?
3. Do you think that salt reduction materials (showing posters, calendars, etc.) are good for the villagers? Can it work? Can it be effective in promoting healthy behavior? What are the factors that can and cannot? What advice do you have for improving these materials (content, form, distribution, posting method, location, frequency, etc.)?
4. How did you organize and implement each health education activity? What problems have been discovered during the implementation of the project? How did you solve it? (Hint: training, materials distribution, health education activities, and interactive activities)
5. What was the impact of local customs and geography (such as geographical location, economic conditions, village dietary, culture, education levels, etc.) on the project intervention?

**Interview guide 3. Villagers**

1. Have you participated in salt-reduction health education activities? Has anyone else in your family participated? If not, what is the reason? If you participated, which ones have you participated in? What left the most memorable impression? Do you think the activities you participate in are useful? What kind of activities will help you and your family more? (Or you and your family would be more willing to participate).
2. If the interviewee is at high risk of CVD, ask if he/she participated in activities, and the opinions, feelings or experience with the activities.
3. Are there any primary school students at home? If so, did this student participate in the "small soldiers for salt reduction" activity? What do you think about the behavior of salt reduction led by primary school students? Could you please give some suggestions for salt reduction?
