# Supplementary material for: A Mixed Methods Process Evaluation of a Clustered-Randomized Controlled Trial to Determine the Effects of Community-Based Dietary Sodium Reduction in Rural China
Source: Front Med (Lausanne). 2021 May 28;8:646576. doi: 10.3389/fmed.2021.646576 (PMC8192799; doi:10.3389/fmed.2021.646576)
Supplement: Supplementary file 3 [file Data_Sheet_3.docx]

Appendix 3. Quotes about receptivity from interviews

| Theme | Sub-theme | Quote |
| --- | --- | --- |
| Health education materials | Satisfaction | “At the beginning of the project, there were many posters of salt reduction everywhere. We often talked about salt reduction at that time. They were very useful.” (Villager)  “Jingles were really great.”( PPI)  “It was valuable to print salt reduction information on the calendar, so people could get education when they saw the date.” (PPO)  “Posters have achieved a significant effect. The most typical example is a store near the road where low sodium salt has been promoted in that village, which was known by the farmer workers in the nearby villages. They always bought several bags of low sodium salt when they passed by.” (PPI) |
|  |  | “The calendar was distributed to every house, it was hung on the wall and we could see it everyday.” (Villager)  “I found that children were interested in those pictures and stories on the calendar, however, the elderly paid less attention to the details. It depends on the targeted population groups.” |
|  |  | “Stickers are very useful on salt containers. When you add salt during cooking, you would see caution for less salt intake.” (Villagers) |
|  | Dis-satisfaction | “The effect of posters pasted outdoors is not as good as those indoors. There are several key places such as the village center, clinics, stores, where posters were easily noticed. Posters pasted outdoors were easily lost and less effective than indoor ones.” (HE)“Posters couldn’t be kept for a long time. They were ripped off by children or knocked off by the wind or moistened by rain. If not updated in a timely way, the effect of the posters were decreased.”（PPC） |
|  | Advice | “In my mind, a slogan printed on the wall is better…eye-catching and direct. Too much detailed information on the poster or other health education materials could not catch villagers’ eyes. Anyhow this information was also included in the lectures given by health educators.” (PPC) |
| Health education activities | Satisfaction | “To create a specific and suitable atmosphere was important at an early stage. And then the subsequent health education activities could be conducted smoothly in various forms.” (PPO) |
|  |  | “I went to the village meeting room to participate in the salt reduction meeting. The village doctor gave us a lecture, told us lot of information about the harms of excessive salt intake and the benefits of less salt intake, and then sent us a lot of health education materials. The activities were very useful, it was widely remembered among our village.”(Villager) |
|  |  | The health related activity was very popular among villagers. The people were crowded in the village meeting room. (Villager) |
|  | Dis-satisfaction | “There was no new ideas added with passing of time. At the end of program, participants came to pick up the free presents not for the activities.” (CHE) |
|  | Advice | “I consider the middle/elderly people and students as the key populations.” (PPG).  “To develop and train some student volunteers by cooperating with the Education Bureau, especially in the rural area, maybe is a good practice. ”(PPG)  “Someone is willing to do so and considers it as an honor. A certificate of merit and visits by the officers could let him feel that what he is doing is meaningful. This is a cost-effective way.” (PPG) |
| Low sodium substitute | Satisfaction | The village doctor told us that low sodium salt is good for our health, preventing high blood pressure and heart disease. I feel very well, so I used low sodium salt. |
|  |  | “We have adapted to the less salty taste after using the low sodium salt for half a year.” (CHE) |
|  |  | “The price is acceptable—Now the economy is developing, we also pay more attention to health.” (Villager) |
|  | Dis-satisfaction | “Obviously, it was less salty even we used two times as much low sodium salt than usual during cooking noodles. It tasted weird.” (CHE)  “The taste of low sodium salt substitute was less salty than regular salt. And the price was 1 Yuan more than regular salt.” (Villagers) |
|  |  | “In rural areas, price is a sensitive topic, especially for old people. If this one was only few-cents-cheaper than that one, people here definitely tended to choose the cheaper one.” (PPC)  “Actually, this kind of salt is expensive. People need time and instructions to change their own life habits.” (PPO) |
