# Supplementary material for: A Mixed Methods Process Evaluation of a Clustered-Randomized Controlled Trial to Determine the Effects of Community-Based Dietary Sodium Reduction in Rural China
Source: Front Med (Lausanne). 2021 May 28;8:646576. doi: 10.3389/fmed.2021.646576 (PMC8192799; doi:10.3389/fmed.2021.646576)
Supplement: Supplementary file 4 [file Data_Sheet_4.docx]

Appendix 4 Quotes about contextual factors from interviews

| Theme | Interview respondent | Quotes |
| --- | --- | --- |
| Contextual factors influencing implementation | Policy support | "There are already several projects about hypertension and basic public health services policy support in our county. There are free medical examinations for people over 65 years old once a year. Therefore, some interventions could be conducted by combining with other projects, which would improve the quality and quantity of interventions.” (PPI)  “When conducting this project, we did learn a lot, and improve the quality of chronic diseases prevention. Our county (Qingtongxia in Ningxia) was recognized as National Chronic Disease Demonstration Zone this year." (CHE) |
|  | Administrative support | The local health bureaus paid much attention and have done a lot of work on this project. They organized the meeting especially for this project several times, and solved problem on a timely basis during the implementation. Therefore, we could conduct the interventions smoothly. ” (PPI)  “We assessed each county’s performance at half a year. The salt reduction program is an important point in the assessment system. We always praised good practices and seldom gave criticism, because more criticism—poorer performance. Once some counties found out that they hadn’t conducted the required work, we communicated with the Director of Health Bureau. Work became easier with good coordination.” (CHE)  "I have an advantage. I am the head of the medical team of this County Health Bureau. This position may have made it easier for me to carry out my work. ” (CHE)  "It is a long time to wait for the funding from this project. Sometimes, we have to pay for some gifst used for this project by ourselves in advance. We were not willing to do this, but there was some administrative order. Under this condition, we just conducted as thought it was an administrative task.” ( THE). |
|  | Staff enthusiasm and contribution | "About 10% of my work time was spent on this project. Because time is limited, and there is too much work. Sometimes the project affected the work, especially when the tasks are burdensome.” (VHE)  “Many of us are part-time in this project, from the county level to the village level health educators. Hence, in most situation, the time for our main work and this project was conflict. A lot of things in this project were probably done very rough.” (THE)  “This project accounted for 30 percent of my total work. For example, as the chief of the Disease Control Section, I was in charge of some other work including the management of infectious diseases, endemic diseases, cause of death monitoring for HIV/AIDS and so on. Therefore, village health educator had no time and energy but conducted health education for this program in the evenings or on weekends. Village health educators always worked overtime.” (CHE)  “Firstly, I tried my best to become familiar with the program protocol and then give right direction to others. The points and problems should be mastered in advance. I addressed these points repeatedly to the local health workers”. (CHE)  “Regarding our conducting a “good cook” competition, I went to the community by myself to meet with village health educators in advance, and then go to see how the competition was conducted. We summarized the experience as a standard practice as a reference for other villages.” (CHE) |
|  | Young adult migration to cities | "There are many middle-aged and young people working in the city. People staying in the village are mostly the elders, who also do some farm work. They had not much time to attend the activities every time". (VHE)  "There are a lot of young people working in the city. Especially during the summer". (VHE) |
| Contextual factors influencing the interventions effect | Behavior change requires gradual adaptation | " After this project, many villagers know that eating excessive salt is not good for their health, but it is a habit problem. It is very hard to change habit established for a long time.” (THE)  "Reducing salt intake actually is to change the dietary behavior of the villagers. They should gradually adapt and change. ".(CHE ) |
|  | Education level | "The education level of villagers is generally low. There takes a much longer time for them to change behavior”. (CHE) |
